# 2382: Qualitative study of CVS risks perception, knowledge, and behavior among hypertensive African-Americans in South Bronx, NY

**DOI:** 10.1017/cts.2017.268

**Published:** 2018-05-10

**Authors:** Maria Espejo, Balaventkatesh Kanna, Erida Castro-Rivas, Shirley Magabo, Tina Washington, Mohammad Faiz, LaShaun Trimble, Namita Tiwari, Euripides Roques, Andrea Faraci, Edgardo Guzman

## Abstract

OBJECTIVES/SPECIFIC AIMS: Compared to others, African-Americans (AA) have a higher prevalence of hypertension. Although, hypertension control has been well studied in clinical settings, a significant number of AA patients have uncontrolled hypertension. We conducted a qualitative study on CVD risk perceptions, knowledge, and behaviors among hypertensive AA in the South Bronx, NY. METHODS/STUDY POPULATION: Hypertensive AA participants, 18 years and older were recruited at a community-based hospital clinic. Focus groups with open-ended questions on CVD knowledge, perception, and behaviors was conducted. Responses were transcribed and transcript was analyzed using open code method. Concepts were formulated, which were then categorized into dominant themes. The sample size was based on the saturation point related to emerging common themes. RESULTS/ANTICIPATED RESULTS: There were 21 patients participated in 3 focus group sessions. The median age was 59 years; BMI median of 31.5 kg/m^2^; 76% were female. In total, 57% had controlled BP and 67% were diagnosed with diabetes mellitus; 8 themes emerged of which unhealthy diet was dominant. Participants acknowledged eating fried foods and meat seasoned with salt contributed to their hypertension. Their food choices were based on family tradition and economical cost more than nutritional value. DISCUSSION/SIGNIFICANCE OF IMPACT: This study reveals that inner city hypertensive AA patients have misperceptions, gaps in knowledge, and barriers to healthy behaviors. We propose to partner with them using shared decision making to raise awareness, knowledge and change in behaviors to prevent CVD in community settings.

